# Development and Pilot Evaluation of a Tablet-Based Application to Improve Quality of Care in Child Mental Health Treatment

**DOI:** 10.2196/resprot.4416

**Published:** 2015-12-30

**Authors:** Kenneth J Ruggiero, Brian E Bunnell, Arthur R Andrews III, Tatiana M Davidson, Rochelle F Hanson, Carla Kmett Danielson, Benjamin E Saunders, Kathryn Soltis, Caleb Yarian, Brian Chu, Zachary W Adams

**Affiliations:** ^1^ Technology Applications Center for Healthful Lifestyles College of Nursing Medical University of South Carolina Charleston, SC United States; ^2^ Ralph H Johnson VA Medical Center Charleston, SC United States; ^3^ National Crime Victims Research and Treatment Center Department of Psychiatry and Behavioral Sciences Medical University of South Carolina Charleston, SC United States; ^4^ Fuzzco Charleston, SC United States; ^5^ Rutgers, The State University of New Jersey Piscataway, NJ United States

**Keywords:** technology, mobile health, child mental health treatment, feasibility test, fidelity, patient engagement, traumatic stress

## Abstract

**Background:**

Children need access to high quality mental health care. Effective treatments now exist for a wide range of mental health conditions. However, these interventions are delivered with variable effectiveness in traditional mental health service settings. Innovative solutions are needed to improve treatment delivery quality and effectiveness.

**Objective:**

The aim of this study was to develop a scalable, sustainable technology-based approach to improve the quality of care in child mental health treatment.

**Methods:**

A tablet-based resource was developed with input from mental health training experts, mental health providers, and patients. A series of qualitative data collection phases (ie, expert interviews, patient and provider focus groups, usability testing) guided the initial concept and design of the resource, and then its refinement. The result was an iPad-based “e-workbook” designed to improve child engagement and provider fidelity in implementation of a best-practice treatment. We are currently conducting a small scale randomized controlled trial to evaluate the feasibility of e-workbook facilitated child mental health treatment with 10 providers and 20 families recruited from 4 local community-based mental health clinics.

**Results:**

Usability and focus group testing yielded a number of strong, favorable reactions from providers and families. Recommendations for refining the e-workbook also were provided, and these guided several improvements to the resource prior to initiating the feasibility trial, which is currently underway.

**Conclusions:**

This study aimed to develop and preliminarily evaluate a tablet-based application to improve provider fidelity and child engagement in child mental health treatment. If successful, this approach may serve as a key step toward making best-practice treatment more accessible to children and families. As various technologies continue to increase in popularity worldwide and within the health care field more specifically, it is essential to rigorously test the usability, feasibility, acceptability, and effectiveness of novel health technology solutions. It is also essential to ensure that patients and providers drive decision making that supports the development of these resources to ensure that they can be seamlessly integrated into practice.

**Trial Registration:**

Clinicaltrials.gov NCT01915160; https://clinicaltrials.gov/ct2/show/NCT01915160 (Archived by WebCite at http://www.webcitation.org/6cPIiQDpu)

## Introduction

### Background

One in four US children experiences a mental health disorder with severe impairment or distress during their childhood [1-3]. Ensuring that these children have access to the highest quality mental health care is a top public health priority. Efficacious child and adolescent treatments exist for a wide range of mental health disorders [4]. However, these treatments are delivered with variable fidelity in mental health service settings, even among well-trained providers [5-10]. Provider fidelity generally refers to the degree to which a clinician adheres to a treatment protocol and delivers the treatment competently [11]. Drifting or deviating from empirically supported treatment protocols can diminish an intervention’s potency and effectiveness, leading to a major quality shortfall [10,12-14]. Statewide and national dissemination and implementation initiatives are underway to narrow these gaps [15-17]. However, the problem of weak and inconsistent provider fidelity persists and must be addressed to improve the quality of care.

### Technology-Based Resources and Access to High Quality Care

Recent technological advances offer an opportunity to support the effective delivery of best-practice interventions. This can be achieved with portable mobile apps that assist providers as they implement treatment activities that are challenging to deliver with high fidelity and child engagement. Technology-based decision support tools have been developed in the broader health care field, and initial data suggest that this approach improves clinical decision making and adherence to best practices and treatment protocols [18-19]. Novel technology-based therapy tools can offer a standardized guiding framework for providers to follow as they progress through a treatment and incorporate several design features to promote provider fidelity to a treatment model, such as (1) inclusion of a diverse yet finite set of in-session and homework activities that are all consistent with the goals of the treatment (ie, rather than off-topic or off-task activities that may encourage drift); (2) presentation of key intervention-related concepts in a consistent manner across providers and clients; and (3) assessment and tracking of progress through a treatment model for each client. Moreover, studies in child education suggest that the integration of interactive games, touch screen learning, video demonstrations, and other engaging features enhance child engagement in learning activities [20-21]. Together, these data increase confidence that technology-based approaches may have value toward improving child engagement and provider fidelity in child mental health treatment. Increased child engagement is particularly important during mental health treatment sessions, because engagement has been shown to reduce risk for dropout, which is another pervasive problem in mental health treatment that limits its impact [22].

Efforts to develop technology-based solutions for mental health care must account for limited resources available in community mental health settings, including the cost and providers’ time [23-25]. Contemporary mobile devices such as tablets and mobile phones are low cost and increasingly ubiquitous [26]. Integration of these devices into practice is therefore likely to be feasible from a cost perspective. With regard to time and effort, it is important that novel solutions are user-friendly and able to be integrated readily into practice with minimal provider training and preparation. This is most likely to be achieved when providers and patients are the key drivers of the development process, working closely with the research team at each phase of design, development, and evaluation. A patient- and provider-centered approach is critical for successful implementation and dissemination.

Research is needed that directs the process of developing novel health care solutions and that measures their potential to improve the quality of mental health care. Research reviews suggest that technology-based tools, broadly considered, effectively enhance mental health care; however, the majority of this research focuses on self-help tools for adults [27-29] or other resources used by patients outside the context of formal treatment sessions [30-32]. Our protocol differs from prior approaches by examining the benefits of mobile device apps used *in session* with an emphasis on interventions designed for children and their caregivers.

### Selection of a Treatment Model With Which to Test a Novel Technology-Based Solution

Selecting an appropriate treatment model is an important step in the process of evaluating a new health technology solution. Ideally, the treatment model should have a strong evidence base and high potential for cross-application with other treatment approaches to enhance generalizability of the data. We selected Trauma-Focused Cognitive Behavioral Therapy (TF-CBT) [33-34]; because it is a well-established treatment for children in the mental health field [33-38], has ample evidence supporting its effectiveness [33-38], is widely used, and has been disseminated internationally. Moreover, TF-CBT uniquely addresses multiple symptom domains commonly encountered in mental health treatment settings, including posttraumatic stress, depressed mood, and disruptive behavior. Development of a tablet-based application, or “e-workbook,” for TF-CBT therefore would appear to have high potential to enhance the relevance of our data to a range of established treatments for youth. TF-CBT also requires caregiver involvement, which will allow us to explore the use of in-session resources with adults as well as children. In our pilot work, we conducted structured 30-min interviews with 21 certified national TF-CBT trainers, which revealed significant provider interest in and likely acceptance of tablet-based aids for delivering TF-CBT. These interviews also provided strong direction around key challenges that tablet-based resources can overcome [39].

### Purpose and Aims

The purpose of this project was to develop and pilot a tablet-based e-workbook that is designed to increase quality of care in child mental health treatment via improvements in child engagement and provider fidelity. The potential impact of an e-workbook approach, if successful, is extraordinarily high in light of its scalability and sustainability. In the current protocol, we describe a strategy to develop an e-workbook to augment delivery of TF-CBT, a well-established treatment for children and their caregivers. If this approach is found to have utility in practice, it can be applied to a wide range of treatment approaches. The general outline of the protocol addresses three aims: (1) to develop an e-workbook to support delivery of treatment with high fidelity; (2) to conduct usability tests of the e-workbook with families and providers; and (3) to conduct a feasibility trial comparing TF-CBT vs iPad-facilitated TF-CBT.

## Methods

### Study Design

The aims of this investigation were accomplished in three phases. Phase I included the initial development of the TF-CBT e-workbook. Phase II included focus groups and individual interviews with 21 providers and 24 children (aged 8-16 years) to inform the refinement of the e-workbook (ie, alpha testing). It also included internal beta testing to guide the final editing and debugging process. Finally, Phase III, which is currently underway, features a feasibility trial with 10 providers and 20 families to examine the feasibility of the methodology that we propose to use in a future randomized controlled trial (RCT) as well as the feasibility and acceptability of implementing the TF-CBT e-workbook in community mental health and child welfare agencies.

### Phase I: Development of the TF-CBT e-Workbook

#### Technical Approach to Development

The e-workbook was developed as a Web-based, rather than native (ie, device-specific) application. Although this approach requires the user to have Internet access, an advantage is that Web-based tools are accessible to providers on any of the wide range of web-connected devices. A native application would ultimately require reprogramming and updating for each operating system (Android, iOS, Windows). Although no technology can be completely “future proof,” it was determined that Web-based tools would have the highest potential to remain useful in meeting the aims of this project as technology progresses. Native versions of the app will likely represent the final step toward disseminating this resource once it is fully evaluated and refined.

The application is responsive; that is, it detects the type of device that is in use and adjusts to it for optimal look and feel. For example, the application can detect an iPad and use JavaScript to convert desktop mouse actions to accelerometer actions. This functionality enables the use of features such as shaking the device to trigger a response, inertial finger drawing, and the built-in microphone. Conversely, those actions would naturally degrade to normal mouse actions if a user were to access the resource via desktop computer. As the application detects different devices, the responsive layout naturally adapts to the devices’ native display parameters. This achieves a fully integrated and device-agnostic application and increases potential for adoption across practice settings.

#### Content Development for the TF-CBT e-Workbook

Qualitative data, collected regularly as part of consultation calls conducted in TF-CBT training programs, provided valuable direction in the technical and content development phases. Specifically, these data were used to determine which components of TF-CBT are most vulnerable to drift and what activities could be developed to overcome challenges to child engagement and provider fidelity in each TF-CBT component. For example, providers who were trained in TF-CBT reported that they used the psychoeducation, anxiety management, and coping components of TF-CBT at least 50% of the time with their child trauma cases; but that the exposure components were reported least frequently (26-50%) [40]. Therefore, greater attention was paid to emphasizing exposure-specific elements of TF-CBT rather than general psychotherapy skills. The research team also carefully reviewed data collected from expert clinicians [39] to identify areas in which technology-based resources would have high potential to enhance (1) provider fidelity; (2) child engagement in treatment activities; (3) child or caregiver understanding of key treatment concepts; (4) likelihood of skill acquisition; or (5) patient adherence to homework exercises. Taken together, these data and observations were used to create the content and format of the e-workbook activities and resources listed in Table 1.

We developed numerous resources (Table 1) or “chapters” for use by providers during individual sessions with each child. Introduction videos were created for children and are available on the first screen of each chapter. Each video depicts a teenager who explains the rationale for the chapter and presents brief examples that illustrate completion of the activity. Our decision to feature older youth (ages 15-16) in most of the videos was based on focus group feedback from children aged 8-15 years. Specifically, youth at the younger end of the age range stated that they would be equally pleased with younger or older actors in the videos, whereas youth at the older end of the age range agreed unanimously that actors should be older adolescents, and that they would likely experience very little connection to a younger actor. Some videos are brief (ie, 30-60 second) clips designed for a provider to use after introducing a concept or teaching a skill with the goal of enhancing engagement and reinforcing what the provider taught during the session (eg, videos demonstrating the CBT triangle in the cognitive coping component of TF-CBT). Some chapters feature interactive touch screen games, such as drag-and-drop activities, drawing tools, trivia-style card games, and animated relaxation activities. Each activity was developed to address an element of the TF-CBT protocol that was identified by experts and providers as challenging to implement with high fidelity and engagement.

**Table 1:** Patient-targeted components of the TF-CBT e-workbook by session.

Treatment concept	TF-CBT e-workbook resource	Modality
One for each component of TF-CBT^a^	Introductory videos that provide an overview for the caregiver and child about why this component of treatment is important.	Video clips featuring adolescent-aged subjects
Each TF-CBT component^a^	Interactive homework assignment checklists with activity suggestions.	Interactive application
Psychoeducation^a^	“What Do You Know?” question and answer quiz game, with “card decks” designed to facilitate child-provider education around trauma, domestic violence, sexual abuse, physical abuse, personal safety, disasters, serious accidents, and bulling/peer victimization; these decks can be personalized to each patient and provider.	Interactive touch screen activity with scorekeeping
Psychoeducation	“You are not alone” interactive graphical display that provides accurate statistics about traumatic events and emotional recovery. The provider selects a question to review with the child, and the child then estimates via touch screen interaction how many of children drawn on the screen have had experiences similar to him/her. Correct answers are given with light up figures.	Interactive touch screen activity
Psychoeducation	“Your Body” cartoon that is designed to facilitate accurate labeling of body parts via drag-and-drop touch screen activity. Both genders are represented in this activity.	Interactive drag-and-drop touch screen activity
Stress management^a^	Narrated, illustrated activity to facilitate controlled breathing exercises (eg, balloon inflating/deflating at pre-set speeds).	Interactive “game” application
Stress management	Narrated, illustrated activity to assist with progressive muscle relaxation. The user touches a muscle group on the screen, the muscle group lights up on the image, and detailed instructions are narrated as the child follows along.	Interactive application
Trauma narrative^a^	Users are presented with a drawing tool where they write and/or draw their narratives using a stylus. Handwritten text and/or illustrations are created, and can be saved or exported.	Interactive drawing application
Affective regulation^a^	This tool includes several interactive activities (eg, writing board, feelings wheel, emotions thermometer) to guide child-provider education regarding emotion identification, emotion intensity, and coping skills.	Videos and touch screen activities
Cognitive coping^a^	This chapter includes a variety of educational tools such as instructional images and video clips to guide learning and provider-child interactions. The cognitive triangle is introduced. Next, children are presented with a series of videos depicting children in a variety of ambiguous situations, and are prompted to identify and discuss with their providers about their thoughts, emotions, and behaviors.	Videos and touch screen activities
In vivo exposures	Illustrated tool that uses audio narration to guide provider-child discussion around development of an exposure hierarchy by choosing exposure activities that are safe, feasible, and relevant. Narrations and illustrations are tailored to child sex and index trauma type.	Illustrated application with audio narration
Enhancing safety^a^	Trivia-style activity to facilitate child-provider education around OK/Not OK touch, managing bullying, help seeking, problem solving skills, spotting danger-signal cues, drug refusal skills, Internet safety, and coping with ongoing stressors. These decks can be personalized to each patient and provider.	Interactive touch screen activity
Conjoint sessions^a^	Homework activities to help the child prepare for conjoint sessions.	Homework activity

^a^This resource was identified by TF-CBT trainers as a necessary component to the toolkit (Hanson et al, 2014).

Some resources included activities for providers to complete with caregivers. These consisted of an extensive collection of video clips with narration that demonstrate a wide range of effective behavior management skills, including common mistakes and how to correct them. Videos were not intended to replace provider instruction and demonstration, but to support providers’ attempts to teach caregivers how to apply skills across a broad range of situations and settings. Additional resources were developed for the provider. These consisted primarily of content that assists with session preparation tasks, such as setting session goals and potential agenda items, tracking, updating assigned homework activities, and guiding their clinical decision making as families progress in TF-CBT. We also created provider tabs for most resources that provide discussion points, tips, and ideas for supplemental exercises.

### Phase II: Alpha and Beta Testing

#### Procedure

The primary purpose of alpha testing in software and intervention resource development is to assess reactions and obtain direct input from end users regarding design, content, and functionality. These data are needed to guide improvements to the resource. Participants were provided with tablets (ie, iPads) in either individual interviews or focus group settings to interact with a select set of resources within the TF-CBT e-workbook. Providers were given access to all activities listed in Table 1, and children were given access to a representative sample of activities. Participants were asked a series of semistructured questions administered by trained interviewers. The questions asked participants to provide reactions to each tool, ranging from reactions to the overall “look and feel” of the interface, ease of navigation, and technical problems they experience. An average of 5 minutes was dedicated to review and discuss each resource. Qualitative data collected during these sessions were audiotaped for transcription, coding, and data analysis.

#### Participants

Alpha testing was completed via focus groups with providers (n=22) and via focus groups or individual interviews with children (n=24). Focus group sizes were generally between 5-8 participants.

Children aged 8-11 years and 12-16 years participated in separate groups; average age was 13.0 years. Children were recruited from clinical sites (local child advocacy and mental health treatment centers) and local schools with on-site mental health services with high rates of trauma exposure. Inclusion/exclusion criteria for clinic-referred children were consistent with those used in prior RCTs with TF-CBT: children were 8-16 years old, had a history of at least one potentially traumatic event, had current clinically elevated symptoms of PTSD, and did not have active suicidal or homicidal ideations or symptoms of psychosis. Participants recruited from the schools were also 8-16 years old but not required to have a reported trauma history or a particular symptom profile. This combined clinical/nonclinical sample was used to maximize input on the look-and-feel of the TF-CBT e-workbook in addition to applicability and clarity of content to youth in the targeted age range. Sample of children was 65% female, 86% Black/African American, 14% white.

Providers were recruited from several child advocacy and mental health centers. Providers were eligible if they were fully trained in TF-CBT and carried active child trauma cases at the time of the study. There was diversity in the provider sample with respect to discipline (64% counseling, 18% social work, 18% psychology), credentials (18% doctorate, 23% MSW/LCSW/LISW/LMSW, 36% LPC, 23% other Master’s degree), and years of experience delivering child trauma treatment (46% 5<years, 27% 3-5 years, 27% 1-3 years). Providers also varied in the settings in which they worked (45% mental health clinic, 55% child advocacy center), with several providers delivering TF-CBT in schools or patients’ homes through outreach clinics. The provider sample was 86% female, 73% Caucasian, 18% Black/African American, 4.5% Native American, and 4.5% other (unspecified).

#### Data Analysis

Focus group and interview responses were audiorecorded and transcribed. First, a content analysis of the interview responses was conducted through multiple close readings of the transcriptions by two independent coders (authors on this paper). Each coder generated an independent list of thematic categories and subcategories based on their review of the data (eg, usefulness of a specific tool; age appropriateness). These themes were then further developed and ordered by the primary coder and reviewed and edited by the second coder. The coders then met in a consensus conference to discuss the categories, resolve questions, and refine the thematic categories. Once this was accomplished, the themes were again reviewed. After additional discussion to review and refine categories and resolve questions, the final thematic categories were completed and higher-order categories were developed. We have previously used similar analytic approaches to qualitative research with a range of public sector health care patient and provider populations. [39,41-43] Results of these analyses, reported in Table 2 (providers) and Table 3 (children), were used to refine and fine tune the TF-CBT e-workbook. The vast majority of recommendations given by children and providers were addressed in the context of the current grant prior to feasibility testing. Some recommendations were cost prohibitive, but can be addressed with future funding in preparation for a large-scale RCT.

**Table 2:** Summary of qualitative feedback from mental health providers (n=22) during alpha testing.

e-workbook component (activity)	Positive feedback (mental health providers)	Recommendations^a^ and observations (mental health providers)
Psychoeducation (What Do You Know? card game)	Liked provider tab	Most providers wanted to organize their own decks
	Activity was clear	Most providers wanted to create their own unique cards
	Liked the game component	One provider asked us to label “explicit content” on cards that are particularly sensitive in nature
	Good for full age range; will increase engagement	Ability to “block” certain cards per patient
	Liked interactivity	Some providers asked for more decks of cards—like cyberbullying and Internet safety questions
Psychoeducation (You Are Not Alone activity)	Difference in race/ethnicity	Include percentages and graphical presentations for older kids
	Would use with kids 8-15 years; very helpful in teaching psychoeducation	Option to view statistics for other gender
	Liked the option to address different traumas	Update one of the statistics
		Include figures with blond and red hair
Psychoeducation (Your Body activity)	Very useful in CSA cases	For some patients, would be nice if figure emphasized mouth and hands
	Likely to engage kids	Figure is not “real-looking”
	Straightforward	
	Would be useful as a homework resource for families	
	Use with (8-10 years)	
Relaxation (breathing/PMR coaching activity)	Great resource to have for families to practice at home	Would not use with older kids (other methods like visualization)
	Use with kids 8-12 years; however, some of them noted that they would use with any age range	Balloon would likely be engaging for younger kids, not for older kids (maybe different graphic like a chest or lungs)
	Good introduction to breathing exercises	Would only use this as intro to breathing and would then practice belly breathing as usual
	Helpful in delivery of treatment	An additional imagery resource for teens could be added (pictures, relaxing sounds)
Affective regulation (Writing Board and Feelings Wheel activities)	Activities are clear	Writing board should be larger; option to type; stylus; different colors
	Feelings wheel is engaging	Feelings Wheel: Intensity scale should be more obvious; add word anchors on scale along with numbers; use faces to assist with intensity scale for younger children
	Some thought good for all ages, some thought only good for ages 8-11 years	Should have more emotions or the option to pick your own emotion
	Activities are all very helpful and engaging	
Cognitive coping (thoughts-feelings-actions activity)	Videos are great	Felt activity was too long/might lose interest
	Activity to identify T-F-A	No need for example—videos are enough
	Like the checkmark	Just have a page with CBT triangle and free-text boxes for the child to practice
	Use with any age range	

^a^Many items in the *recommendations* column already have been addressed by the development team. Recommendations made by several providers, such as the ability for providers to organize decks and create new cards in the “*What Do You Know?”* game and the recommendation to add decks addressing Internet safety and cyberbullying, were addressed by the developers in the revised e-workbook prior to the feasibility trial. Other recommendations were only voiced by one or two participants, and were considered on a case-by-case basis. Some observations made by providers related to child age; however, these observations often contradicted the perceptions of children. For example, some providers felt that two or three of the chapters were best for children under 12 years of age; but in each of these cases older adolescents (aged 13-16 years) responded very favorably to the chapters. We have informed providers of this feedback from adolescents and have asked providers to be open-minded in the context of the feasibility trial about the value of each resource on the basis of age.

**Table 3:** Summary of qualitative feedback from children (n=24) during alpha testing.

e-workbook component (activity)	Positive feedback (children)	Recommendations^a^ and observations (children)
Psychoeducation (What Do You Know? card game)	Using technology would help kids be more comfortable	Audio narration that reads card to you
	Most youths said that they would like the iPad-based version over the real cards	Too much white background—include more colors and enhance flexibility
		Add more colors or brighter colors
		Have themes or animation that relates to the question OR that the kid can choose to “make it their own”
Psychoeducation (You Are Not Alone activity)	N/A—Children were not asked about this activity	
Psychoeducation (Your Body activity)	N/A—Children were not asked about this activity	
Relaxation (breathing/PMR Coaching activity)	Activity makes sense	Balloon is fine, maybe integrate other graphics, like lungs, or a kid breathing in and out
	Would be helpful to learn to calm down	Change white screens to something else—have options for different background colors (preferred neon)
	App version is very straightforward	Make activity available to kids/teens at home
	Like that they can practice many times	Drawn images of children should look more like a real person
	Appropriate for full age range of youth	Have a total body video
		Have buttons in order of muscle groups
		Make girl look older
Affective regulation (Writing Board and Feelings Wheel activities)	Like the activities	Want the option to type; use different colors; make screen look like notebook paper/chalkboard
	Engaging	Have the wheel make spinning noises
	Majority would want to use tech version instead of doing these activities with paper and pencil	Do not know some words (eg, elated)
	Like the feelings of charades game	Use faces as anchors on intensity scale
		Add additional link if the kid/teen wants to learn more about something
Cognitive coping (Thoughts-Feelings-Actions activity)	N/A—Children were not asked about this activity	

^a^Many items in the *recommendations* column already have been addressed by the development team. Recommendations made by several providers, such as the ability for providers to organize decks and create new cards in the “*What Do You Know?”* game and the recommendation to add decks addressing Internet safety and cyberbullying, were addressed by the developers in the revised e-workbook prior to the feasibility trial. Other recommendations were only voiced by one or two participants, and were considered on a case-by-case basis. Some observations made by providers related to child age; however, these observations often contradicted the perceptions of children. For example, some providers felt that two or three of the chapters were best for children under 12 years of age; but in each of these cases older adolescents (aged 13-16 years) responded very favorably to the chapters. We have informed providers of this feedback from adolescents and have asked providers to be open-minded in the context of the feasibility trial about the value of each resource on the basis of age.

**Figure 1:**
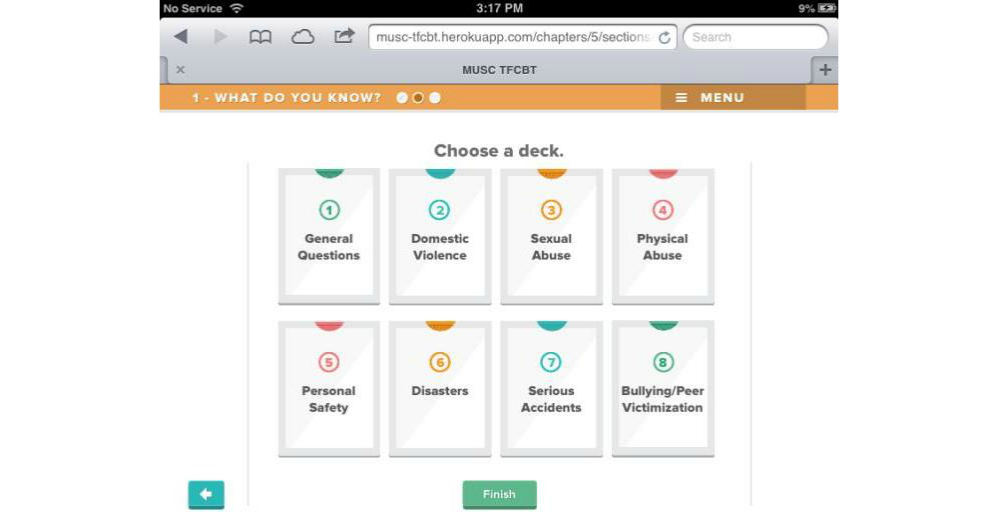
Screenshot of the eight “decks” of virtual cards in the “What Do You Know?” psychoeducation activity. Clinicians and families select the most pertinent decks for each child. The cards within each deck are customizable by the clinician to enhance relevance to each family’s needs.

**Figure 2:**
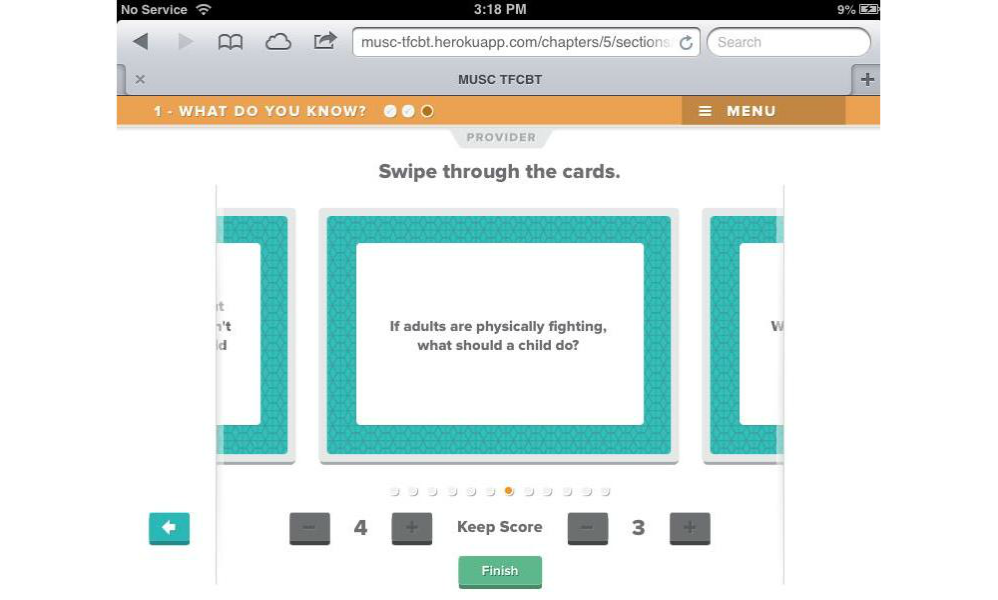
An example card from the Domestic Violence deck. Users can swipe left or right through the deck to view additional cards. A scoreboard is presented at the bottom to increase engagement.

**Figure 3:**
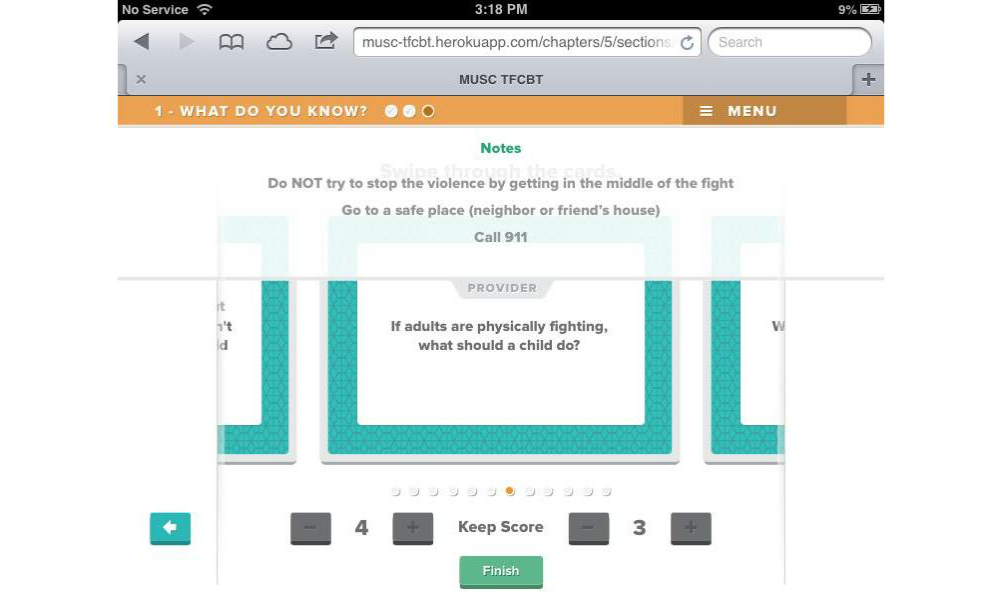
A Provider tab is located at the top of the screen and contains specific notes for each card. These notes may be used as discussion points during session.

**Figure 4:**
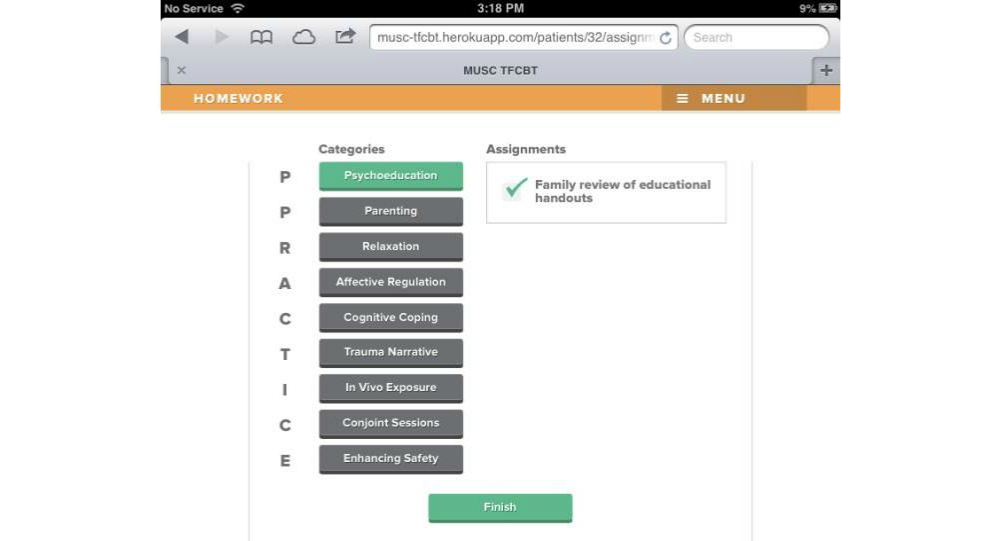
At the end of each activity, progress is tracked graphically via color-coding (ie, green=complete). On this page, module-specific homework assignments may be assigned. The Menu button at the top of each screen allows users to access the Home screen or end a session and assign homework.

**Figure 5:**
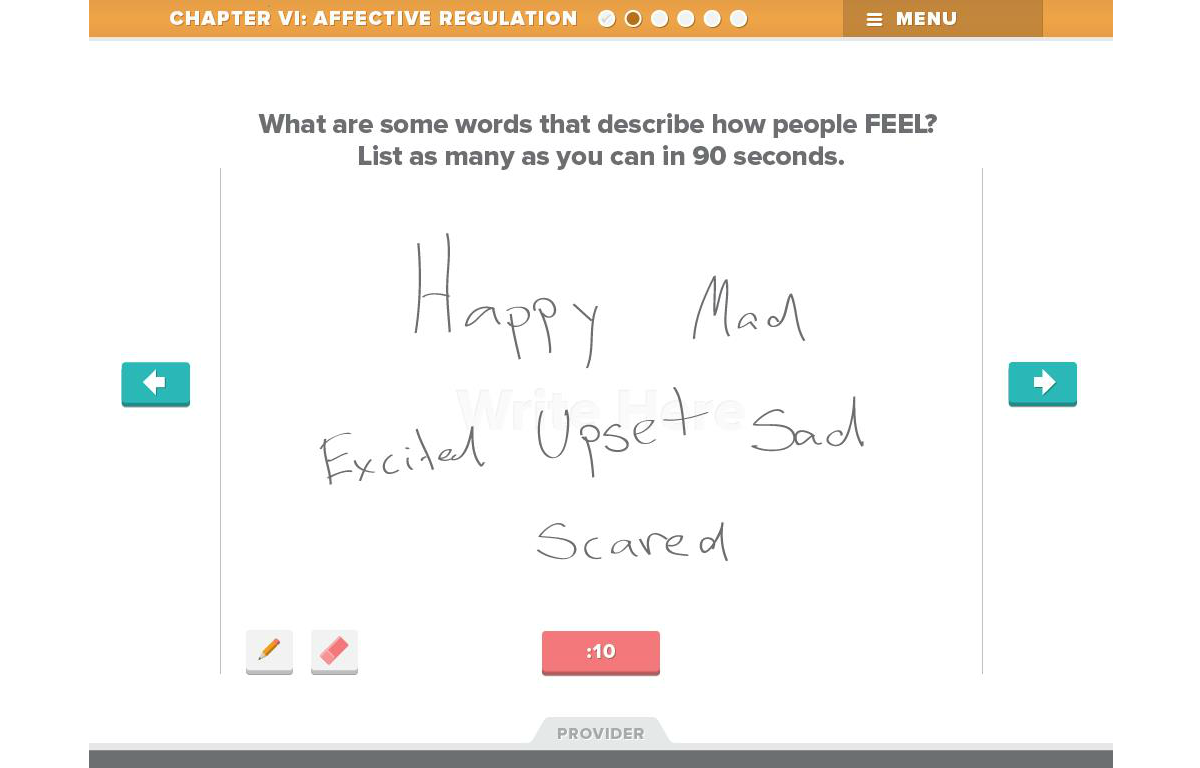
One activity in the Affective Regulation module involves directing the child to list as many different feeling words in 90 seconds as he or she is able to. This activity uses a free-write function. The Provider tab at the bottom of the screen provides tips for clinicians on strategies for using this activity effectively in session.

**Figure 6:**
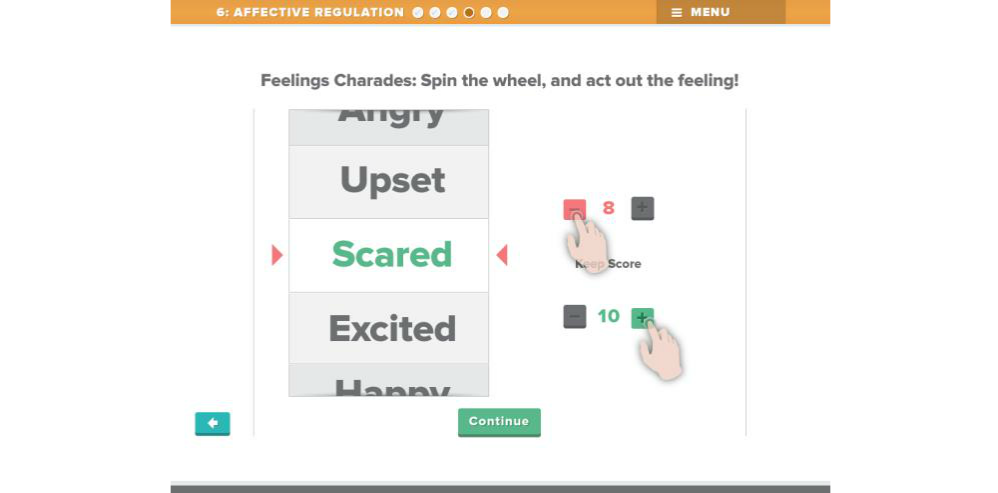
Another Affective Regulation activity is Feelings Charades. Users “spin” a virtual wheel by dragging down on the feeling words. They are then instructed to act out the feeling on which the wheel lands. A scoreboard is available to increase engagement with the activity.

**Figure 7:**
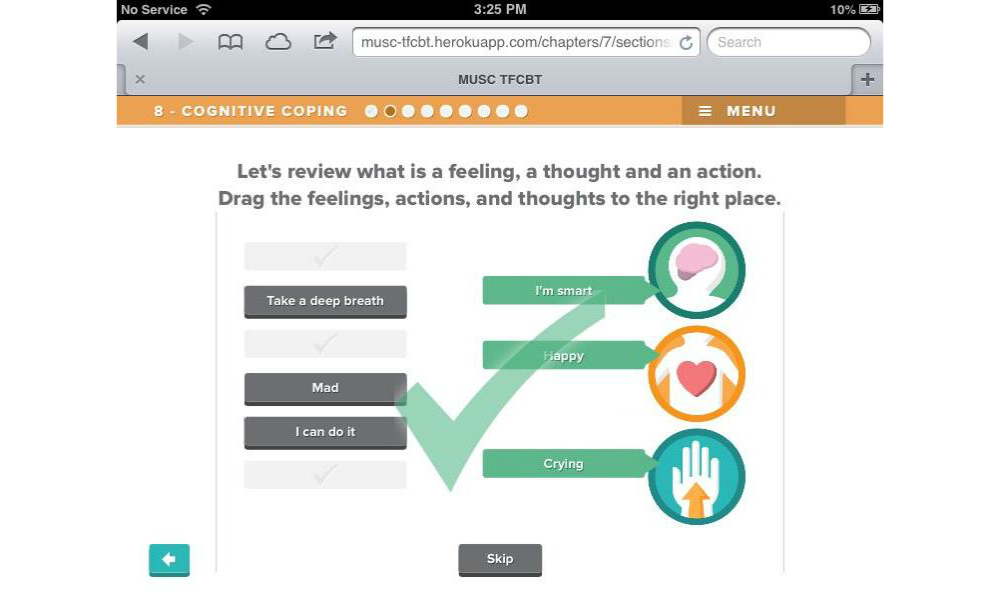
An activity in the Cognitive Coping module has children practice identifying and categorizing thoughts, feelings, and behaviors by dragging the words and phrases on the left of the screen (shown in gray) to the appropriate icon on the right of the screen. Feedback is provided in response to each user interaction.

Beta testing followed the alpha testing phase and is a form of usability testing in which refined or revised resources are evaluated by a new set of providers for acceptability and functionality. This was to ensure that any errors existing within the TF-CBT e-workbook were identified and corrected in preparation for the feasibility trial. Members of the development and investigative team reviewed all the revised components of the resource, and additional edits and refinements to content, appearance, and functionality were made in response. Figures 1-7 include screenshots of several e-workbook activities.

### Phase III: Feasibility Trial

#### Approach

The goal of the ongoing feasibility trial is to examine the feasibility of implementing the TF-CBT e-workbook in community mental health service agencies, and to demonstrate the randomized controlled trial (RCT) methodology we propose to use in a future large-scale trial to test the effectiveness of the TF-CBT e-workbook in clinics across the state. Resources and data resulting from this project are designed to position us well for a large-scale RCT to examine the incremental benefit of e-workbooks to best-practice interventions compared to standard care without technology. The strengths and limitations associated with conducting a small-scale feasibility RCT versus an open trial were carefully considered. A major benefit of the latter approach included increased quantity of feasibility data related to the use of the e-workbook. On balance, this option was outweighed by the importance of demonstrating the feasibility of the full RCT methodology. This would allow us to identify and address barriers in recruitment, retention, fidelity assessment, and other procedures essential to successful conduct of a RCT. This approach is consistent with expert recommendations to pilot test the feasibility of methods to be used in large RCTs, use data yielded by such studies to “debug” the methodology, and assess optimal strategies to executing the RCT [44].

#### Design

The feasibility trial will involve 10 providers recruited from four participating community-based mental health and child welfare agencies where TF-CBT is delivered. Providers will have been fully trained in TF-CBT and carry active child trauma cases at the onset of the trial. Providers will be block randomized by site to either the e-workbook facilitated TF-CBT (n=5) or standard TF-CBT (TAU) (n=5) conditions. Each provider will treat at least two children for a total sample of 20 children. We will also pursue an exploratory aim regarding the feasibility of using the e-workbook across multiple treatment delivery settings by including a small subsample of mental health providers who are primarily based in school settings (at least two per study condition).

An independent, trained evaluator who is blind to the study condition will conduct baseline, mid-treatment, and post-treatment clinical assessments. Families will be reimbursed $30 for each administration of the assessment battery. All sessions will be audiorecorded and coded for fidelity and child engagement by independent coders.

Alternatives to this design decision were considered carefully. For example, the design could have included each provider treating one e-workbook case and one standard TF-CBT case, with randomized ordering of conditions by provider. An advantage of this approach is that provider factors are less likely to have strong impact on clinical outcomes in this small-scale trial. A weakness of the approach is that use of the e-workbook could affect performance on subsequent standard TF-CBT cases for providers assigned to deliver TF-CBT using the e-workbook first. Additionally, it was determined that if providers within an agency were assigned to different starting conditions, this could increase risk of contamination across conditions with standard TF-CBT providers hearing about, viewing, and potentially even using elements of the e-workbook with standard TF-CBT cases. Overall, it was concluded that block randomization by clinic to the two study conditions was a preferable design strategy.

#### Participants

##### Providers

A minimum of 10 providers who have been trained in TF-CBT and maintain active child trauma caseloads will be recruited from four participating clinics in the Charleston tri-county area. The clinics include both outpatient mental health clinics and child advocacy centers. We will make every effort to recruit a racially/ethnically diverse sample of providers to allow integration of feedback from providers with diverse backgrounds. We will also attempt to recruit a balanced sample with respect to sex and gender. Inclusion criteria for providers will be as follows. Providers will be full- or part-time employees of the participating clinic and had to have obtained at least a Master’s degree in social work, counseling, clinical psychology, or a related field. Each provider will provide treatment to at least two study cases during the feasibility trial. Providers will be encouraged to enroll up to 5 children to increase the likelihood that at least 2 cases will be completed per provider during the trial.

##### Patients

A minimum of 20 children aged 5-15 years and their caregivers will be recruited into the feasibility trial. These children will be recruited from participating clinics in the local area. We will make every effort to recruit a racially/ethnically diverse sample of youth (eg, >25% African American) to ensure integration of feedback from patients with diverse backgrounds. Inclusion criteria will be as follows. Participating children will be aged 5-15 years, have experienced at least one potentially traumatic event (eg, sexual assault, physical assault, witnessed violence, disaster, serious accident), and have at least one symptom on each PTSD symptom cluster (re-experiencing, avoidance, hyperarousal) based on a diagnostic interview. Cases will be excluded when the child or caregiver exhibits psychotic symptoms (eg, active hallucinations, delusions, impaired thought processes); significant cognitive disability, developmental delays, or pervasive developmental disorder; or active suicidal or homicidal ideations. Children also will be excluded when there is no consistent caregiver available to participate (eg, short-term foster care placement, restrictions by child protective services). These criteria are consistent with those used in prior TF-CBT clinical trials.

#### Assessment

A trained evaluator both blinded to treatment condition and fully trained in the administration of all measures will administer the assessment battery. All measures are well-validated and widely used instruments in the mental health field and in the treatment outcome research. Assessment will occur on three occasions: at baseline (ie, during the screening interview), at mid-treatment (ie, after the sixth treatment session, approximately two months post-baseline), and at post-treatment (ie, after the twelfth treatment session, approximately four months post-baseline). Treatment fidelity and child engagement will be measured via observational coding of audiotapes.

#### Treatment Fidelity

Fidelity to the TF-CBT and e-workbook-facilitated TF-CBT protocols will be measured via coding of audiotaped treatment sessions by independent, trained raters. Treatment sessions will be audiotaped for both study conditions (n=20 x ~10 sessions average=~200 audiotapes). Ratings will be completed using a behaviorally specific coding system of TF-CBT provider behavior that was modified for the current study to ensure relevance to the e-workbook condition [45]. The coding system will be used to calculate providers' fidelity to each TF-CBT component. An additional eight items focus on general therapy skills, not specific to TF-CBT, including establishing an agenda, providing a treatment rationale, and assigning homework. Additional items were created to identify use of the e-workbook activities to differentiate the two study conditions. Two independent raters will listen to audiorecorded treatment session tapes and complete the modified fidelity measure to code the use and extensiveness of specific treatment techniques depicted on the recordings. Raters will be trained in the coding system and meet biweekly throughout the remainder of the feasibility trial to ensure maintenance of acceptable levels of accuracy and interrater reliability. Discrepant ratings will be reviewed until consensus is achieved. If the two raters cannot reach consensus, the PI and Co-Is will make final decisions.

#### Child Engagement

Child engagement will be measured via coding of audiotaped sessions by independent, trained raters. The Child Involvement Ratings Scale (CIRS) [46-47], a 6-item scale that measures child engagement for each session, will be used for this purpose. Four “positive” involvement items and two “negative” involvement items are rated for each session on a 6-point scale (ie, “not at all” to “a great deal present”). The positive-involvement items emphasize the extent to which children initiate discussions, demonstrate enthusiasm, self-disclose, and demonstrate understanding. Negative-involvement items address withdrawal or avoidance in treatment. Coders will provide ratings based on two 10-min segments of session audiotapes (ie, beginning at min 10 and min 40). Child engagement ratings on the CIRS have been associated with clinical outcomes [47] and provider flexibility in delivery of EBTs [48]. Excellent internal consistency and strong interrater reliability have been reported for this measure [47-48].

#### Child-Report of Functioning

##### Clinical Interview

The Kiddie Schedule for Affective Disorders and Schizophrenia for School Age Children-Present and Lifetime version (K-SADS-PL PTSD module) [49] is a semistructured interview that will be used to assess PTSD symptom levels and diagnostic status. The K-SADS-PL is well-established and used widely. It also has been used in numerous TF-CBT RCTs [33]. The K-SADS-PL also assesses functional impairment in school, social, and family life.

##### Self-Report Instruments

The Center for Epidemiological Studies Depression Scale for Children (CES-DC) assesses the severity of depressive symptomatology in children. The CES-DC is a 20-item self-report depression inventory with possible scores ranging from 0-60. Scores over 15 are indicative of significant levels of depressive symptoms [50,51]. The UCLA PTSD Index for Diagnostic and Statistical Manual of Mental Disorders, Fourth Edition (DSM-IV), Child Version (UCLA-PTSD) [52-54] will be used to assess the severity of PTSD symptomatology in children. The UCLA PTSD assesses exposure to traumatic events and all 17 DSM-IV symptoms of PTSD. Psychometric research has yielded significant support for the reliability, construct validity, and PTSD criterion-related validity [54]. The Therapeutic Alliance Scale for Children (TASC) [55-57] will be used to measure levels of therapeutic alliance. The TASC is an 8-item measure of the child’s alliance with the provider using a 4-point scale. It has good internal consistency and interrater reliability [57]. The Child/Adolescent Satisfaction Questionnaire (CASQ) [58] is a 15-item instrument that assesses child satisfaction with mental health treatment.

#### Caregiver Report of Functioning

##### Clinical Interview

The K-SADS-PL [49] also will be administered to caregivers to assess children’s PTSD symptoms and functional impairment.

##### Caregiver Report Instruments

The UCLA PTSD Index for DSM-IV, Parent Version [59] will be used to assess the severity of the child’s PTSD symptomatology from the perspective of the caregiver. The Child Behavior Checklist-Parent Report (CBCL) [60] is a widely used measure of behavioral and social maladjustment in children, as perceived by the caregiver. The CBCL has strong psychometric properties. The Beck Depression Inventory (BDI) [61-62] will be used to assess severity of caregivers’ depressive symptomatology. The BDI is a 21-item self-report scale of depression and is widely researched and has excellent concurrent and discriminant validity [63]. The Caregiver Satisfaction Questionnaire (CSQ) [58] is a 15-item instrument that assesses caregiver satisfaction with mental health treatment. The Working Alliance Inventory (WAI-short form) [64] is a 12-item measure of the parent-therapist alliance using a 7-point scale (ie, “never” to “always”).

#### User Reactions to TF-CBT e-Workbook

To obtain direct input on the e-workbook’s design, content, and functionality, post-treatment assessments will include semistructured interviews with children, caregivers, and providers after completion of e-workbook-facilitated TF-CBT. Patients and providers will complete semistructured interviews about their experiences with the e-workbook and how it affected treatment. Interviews will be audiotaped, transcribed, and interpreted using the same approach used during alpha testing.

#### Data Analysis Plan

For both conditions, several patient- and provider-level variables, as well as data collection procedures will be assessed and described. Patient-level variables will include the percentage of eligible patients recruited, treatment attrition, study retention, and session attendance. Recruitment will be assessed by the proportion of patients who agree to participate as compared to the total number solicited to enroll. Attrition will be assessed by examining the proportion of patients who prematurely terminate treatment. Qualitative analyses will be conducted to identify themes in termination. Study retention refers to the proportion of patients who complete all assessment points associated with the treatment protocol, including those who terminate treatment prematurely.

Provider-level variables will include provider recruitment to participate, fidelity to TF-CBT procedures, use of the resources within the e-workbook, and adherence to the session audiotaping protocol. Recruitment will be assessed by the proportion of providers who agreed to participate as compared to the total number approached. Provider fidelity will consist of the proportion of completed treatment components for the intervention according to the fidelity measure described previously. Providers will be interviewed at the end of treatment with e-workbook cases to provide feedback on the usability of the e-workbook activities. Other data to be summarized include kappa coefficients between independent fidelity raters, scheduling and logistical barriers to completing assessment, and communication successes and failures between study staff and clinic site staff around recruitment efforts to demonstrate feasibility of study procedures for our planned RCT.

## Results

At the time of manuscript submission, Phases I and II of the study (mobile application development and refinement) were completed, and initial recruitment for Phase III (feasibility trial) is underway. No data have been cleaned or analyzed for the feasibility trial component of the project. All aspects of this federally funded study have been approved by the institutional review board (IRB) at the institution where the research is being conducted. Usability and focus group testing yielded a number of strong, favorable reactions from providers and families. Recommendations for refining the e-workbook also were provided, and these guided several improvements to the resource prior to initiating the feasibility trial, which is currently underway.

## Discussion

The current protocol advances methods for developing technologies for use in mental health. First, it moves the field forward by developing and evaluating technology-based tools designed specifically to support treatment delivery and quality of care by targeting provider fidelity and, second, through engaging children. This represents a key step toward making EBTs more accessible to children and families. Existing technology-based tools largely target the (adult) patient directly by assisting them in self-care or homework adherence [25-43,63]. Such resources are not designed to support providers in the delivery of interventions with fidelity, and therefore are unlikely to have significant direct effect on the quality of care that families receive when they present for treatment in community mental health service agencies. Second, this line of research will provide valuable data about the potential for technology-based resources to enhance children’s engagement in treatment. This is an important, but significantly underdeveloped, area of research that may have critical implications for the health care field. Third, we capitalized on recent technological advances by developing Web-based applications that are optimized for mobile devices. This allows us to test the resources on a tablet (eg, iPad) while ensuring that we achieve a fully integrated and device-agnostic application that will operate with the most current technologies without the need to rewrite the application on a device-by-device basis. Fourth, we developed a wide range of tools (eg, videos, interactive games, drawing applications) that providers will use with children and adults (caregivers). This will ensure collection of valuable feasibility data that have high relevance to several design formats and target populations. Alternatively, a narrower focus on a specific patient population (eg, adults with depression) or specific type of resource (eg, videos only) would have had relatively less potential to significantly advance the field.

This study represents a unique opportunity to capitalize on the increasing use of mobile phone, Internet, and tablets by both patients and providers to support providers’ efforts to deliver empirically supported treatments with a high level of fidelity. As these technologies continue to increase in popularity worldwide and within the health care field more specifically, it is essential to rigorously test the usability, feasibility, acceptability, and effectiveness of these resources. It is also essential to use input from patients and providers to drive decision-making related to development of these resources to ensure that they can be seamlessly integrated into practice. This study takes an initial step toward evaluating the feasibility and utility of implementing best-practice treatment with the assistance of a patient- and provider-centered tablet-based e-workbook resource. If feasibility is supported by our pilot trial, we will propose a rigorous, statewide efficacy evaluation powered to examine the impact on child mental health outcomes. Data yielded from such an evaluation will have tremendous value for purposes of developing and disseminating highly accessible resources that are designed to enhance the quality of treatment delivered to children and families in a wide range of service settings.

## Abbreviations

BDI: Beck Depression Inventory
CASQ: Child/Adolescent Satisfaction Questionnaire
CBLC: Child Behavior Checklist
CBT: cognitive behavioral therapy
CES-DC: Center for Epidemiological Studies Depression Scale for Children
CIRS: Child Involvement Ratings Scale
CSQ: Caregiver Satisfaction Questionnaire
DSM-IV: Diagnostic and Statistical Manual of Mental Disorders, Fourth Edition
EBT: evidence-based treatment
e-workbook: tablet-facilitated Trauma-Focused CBT (mobile application)
IRB: institutional review board
KSADS-PL: Kiddie Schedule for Affective Disorders and Schizophrenia for School Age Children-Present and Lifetime version
PTSD: posttraumatic stress disorder
RCT: randomized controlled trial
TAC-R: Treatment Adherence Checklist-Revised
TASC: Therapeutic Alliance Scale for Children
TAU: treatment-as-usual
TF-CBT: Trauma-Focused Cognitive Behavioral Therapy
UCLA-PTSD: UCLA PTSD Index for DSM-IV
WAI: Working Alliance Inventory
